# The role of metal ions in the occurrence, progression, drug resistance, and biological characteristics of gastric cancer

**DOI:** 10.3389/fphar.2024.1333543

**Published:** 2024-02-02

**Authors:** Pengtuo Xiao, Changfeng Li, Yuanda Liu, Yan Gao, Xiaojing Liang, Chang Liu, Wei Yang

**Affiliations:** ^1^ Department of Endoscopy Center, China-Japan Union Hospital of Jilin University, Changchun, China; ^2^ Department of Immunology, College of Basic Medical Sciences, Jilin University, Changchun, China

**Keywords:** metal ion, gastric cancer, calcicoptosis, ferroptosis, cuproptosis, drug resistance

## Abstract

Metal ions exert pivotal functions within the human body, encompassing essential roles in upholding cell structure, gene expression regulation, and catalytic enzyme activity. Additionally, they significantly influence various pathways implicated in divergent mechanisms of cell death. Among the prevailing malignant tumors of the digestive tract worldwide, gastric cancer stands prominent, exhibiting persistent high mortality rates. A compelling body of evidence reveals conspicuous ion irregularities in tumor tissues, encompassing gastric cancer. Notably, metal ions have been observed to elicit distinct contributions to the progression, drug resistance, and biological attributes of gastric cancer. This review consolidates pertinent literature on the involvement of metal ions in the etiology and advancement of gastric cancer. Particular attention is directed towards metal ions, namely, Na, K, Mg, Ca, Fe, Cu, Zn, and Mn, elucidating their roles in the initiation and progression of gastric cancer, cellular demise processes, drug resistance phenomena, and therapeutic approaches.

## 1 Metal ion: a weight on the scale of health and disease

Gastric cancer, ranking fifth in incidence and third in mortality among all cancers, poses a significant global health burden, causing approximately 800,000 deaths annually (Sung et al., 2021). Notably, variations in gastric cancer incidence are observed across populations, with the highest rates documented in East Asian populations. Chronic infection with the bacterium *H. pylori* (*Helicobacter pylori*) stands as the principal risk factor for gastric cancer, accounting for an estimated 90% of cases (Suzuki et al., 2009), while other risk factors include diet, smoking, and excessive alcohol consumption (Machlowska et al., 2020). Approaches to gastric cancer treatment encompass a range of options. Surgical resection entails the excision of the cancerous region of the stomach along with adjacent lymph nodes and tissues (curative gastrectomy). Furthermore, therapeutic strategies encompass intraluminal stent placement, intraluminal laser therapy, chemotherapy, radiotherapy, and targeted therapy (Petrillo and Smyth, 2020). Nevertheless, these treatment modalities are not without limitations. A retrospective study conducted in Japan involving 118,367 gastric cancer patients who underwent surgical treatment revealed a 5-year survival rate of 71.1% (Katai et al., 2018). Although neoadjuvant chemotherapy has shown promise in enhancing the R0 resection rate and reducing tumor staging, it does not confer a significant advantage in terms of long-term survival rates (Mirza et al., 2013). Immunotherapy primarily serves as a salvage treatment for patients who are ineligible for surgery. While it has demonstrated effectiveness in improving 3-year survival rates, overall survival rates remain relatively low (Boku et al., 2021). Non-surgical approaches to tumor treatment aim to induce apoptosis or necrosis of tumor cells. Unfortunately, tumor cells exhibit significant resistance to these processes, which is believed to be associated with the accumulation of metal ions within the tumor microenvironment, as suggested by current research.

Multiple metal ions found within the human body exhibit specific functions in preserving cellular structure, regulating gene expression, and catalyzing enzymatic activity. These ions also participate in modulating relevant pathways during different forms of cell death. Recent research indicates the presence of numerous ion irregularities within tumor tissues, which are closely associated with the malignant characteristics of tumors. Metal ions exert influence over cellular processes such as proliferation, apoptosis, migration, differentiation, and angiogenesis. Among these processes, extensive investigations have been conducted to understand the regulatory role of ions in cell death. Cell death is of paramount importance for the organism and encompasses well-established pathways like apoptosis (D’arcy, 2019), necroptosis (Yu et al., 2021), autophagy (Mizushima and Komatsu, 2011), as well as newly discovered pathways such as ferroptosis (Li et al., 2020) and cuproptosis (Tsvetkov et al., 2022), all demonstrating the involvement of metal ions. During various stress responses, cell death can occur through one or multiple forms, and the loss of control over single or mixed types of cell death can result in the development of severe diseases, including cancer. The relationship between metal ions and cancer has gained increasing attention in recent years, with both essential elements (e.g., potassium, sodium, calcium, and magnesium) and trace elements (e.g., iron, copper, zinc, nickel, and cobalt) shown to exert varying degrees of influence on different types of cancer.

Currently, metal ions have a significant impact on inducing cell death, including calcium ion-mediated mitochondrial death, iron ion-mediated ferroptosis, and copper ion-related cuproptosis. Calcium ion-related mitochondrial death represents the classical caspase-dependent mitochondrial apoptotic pathway (Jeong and Seol, 2008). Notably, the development of BH3-mimetic drugs targeting the mitochondrial apoptotic pathway has exhibited substantial progress in the treatment of various cancers (Diepstraten et al., 2022). Ferroptosis is a novel form of cell death characterized by iron-dependent accumulation of lipid peroxides. It is primarily induced by iron overload and reactive oxygen species-dependent lipid peroxidation (Li et al., 2020). Currently, research on ferroptosis in tumors mainly focuses on eliminating residual or drug-resistant cancer cells, which may provide new avenues for anti-tumor therapy (Mou et al., 2019). Cuproptosis, a recently discovered form of cell death associated with copper ions, is triggered by the disruption of copper ion homeostasis. Copper death occurs through the direct binding of copper with lipid components of the tricarboxylic acid cycle, resulting in protein toxic stress and subsequent loss of iron-sulfur proteins, ultimately leading to cell death. Moreover, the imbalance of Cu + homeostasis leads to the loss of intracellular iron-sulfur proteins and elevated expression levels of HSP70, further inducing protein toxic stress. These two pathways collectively contribute to cell death mediated by copper ions (Tsvetkov et al., 2022).

**FIGURE 1:**
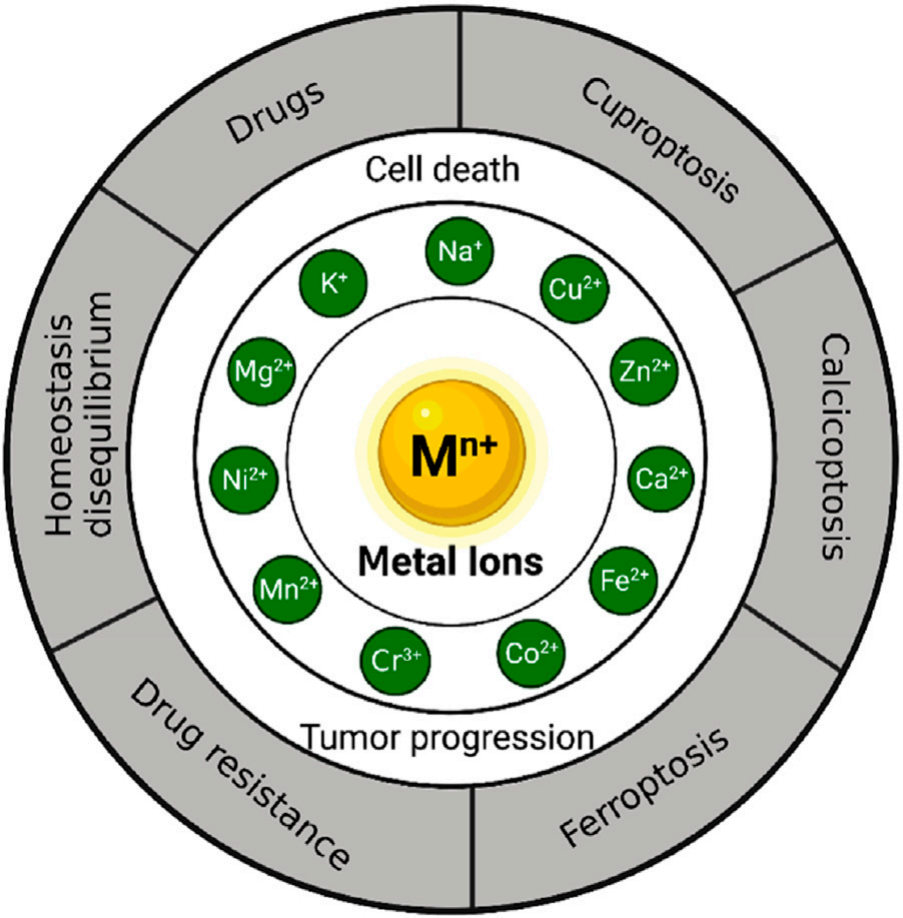
The role of metal ions in the development, cell death and treatment of gastric cancer.

The principal objective of this study entails conducting an extensive literature review on the role of metal ions in the development and progression of gastric cancer. Specifically, this investigation aims to explore the effects of various metal ions, such as sodium (Na^+^), potassium (K^+^), magnesium (Mg^2+^), calcium (Ca^2+^), iron (Fe^2+^,Fe^3+^), copper (Cu^2+^), zinc (Zn^2+^), manganese (Mn^2+^), cobalt (Co^2+^), chromium (Cr^3+^), and nickel (Ni^2+^), in relation to gastric cancer development, cellular apoptosis, and therapeutic interventions (see Figure 1).

## 2 Unveiling the impact of metal ions on gastric cancer

### 2.1 Sodium

Sodium (Na^+^), an essential ion for maintaining physiological homeostasis, has been implicated in the etiology and progression of gastric cancer, as evidenced by numerous investigations. Cumulatively, heightened sodium intake has been linked to an augmented risk of gastric cancer. Epplein et al. (2014) examined the association between dietary sodium intake and the susceptibility to *Helicobacter* pylori-related gastric cancer, revealing a noteworthy increase in the risk of gastric cancer when sodium intake surpassed 3,506 mg/day in the presence of *H. pylori* infection. Conversely, in the absence of *H. pylori* infection, excessive sodium intake alone did not significantly contribute to gastric cancer development. Sufficient quantities of sodium ions activate the destructive activity of *H. pylori* on the gastric mucosa, resulting in ulceration, inflammation, and ultimately the development of gastric cancer. Similarly, Salvador et al. (2015) identified high sodium intake as a significant factor contributing to gastric cancer. Their research indicated that when salt consumption exceeded the recommended level (5 g/day) by 50%, an excessive amount of sodium ions caused damage to the gastric mucosa, thereby becoming a high-risk factor for gastric cancer.

**FIGURE 2:**
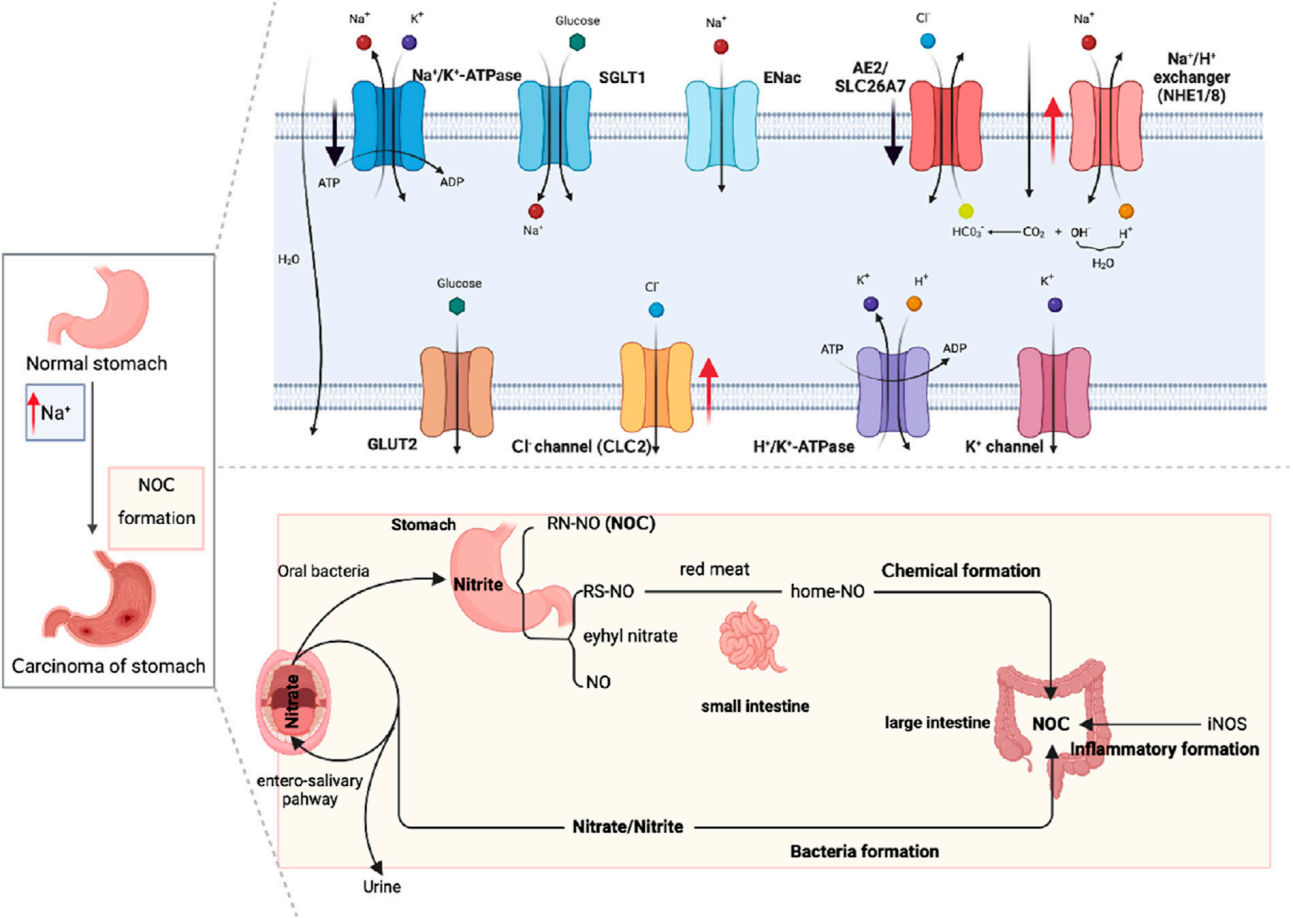
Diagram of sodium ion channels in normal gastric tissue and NOCs induced by high sodium promoting gastric cancer.

Regarding its underlying mechanism, elevated sodium ion levels have been found to heighten gastric acidity, thereby facilitating the interaction between nitrite and amines and promoting the formation of N-nitroso compounds (NOCs) (Jain et al., 2020), which have been demonstrated to induce various cancers, including gastric cancer (Kazmierczak-Siedlecka et al., 2022). In a study conducted by Xu et al. (2015) involving 18,244 patients with gastric cancer, it was observed that these patients exhibited elevated sodium ion levels, and their endogenous NOC levels exhibited a positive correlation with sodium ion levels. This suggests that sodium creates a conducive environment for the formation of NOCs, thereby promoting the occurrence of gastric cancer (Figure 2).

In the realm of signaling pathways, gastric cancer cells experience various modifications in sodium-related pathways. Epithelial sodium channels (ENaC), which are transmembrane proteins responsible for regulating sodium absorption, have exhibited associations with the proliferation and metastasis of multiple cancer types (Liu et al., 2016). Intracellular sodium influx through ENaC has been identified as an intracellular signal preceding cell migration (Kapoor et al., 2009), and the depolarization induced by ENaC contributes to cellular proliferation by impacting the cell cytoskeleton (Chifflet et al., 2005). Moreover, ENaC functions as a mechanosensor, engaging with the cell cytoskeleton and extracellular matrix, thereby generating mechanically gated sodium influx and initiating secondary signal transduction pathways (Wei et al., 2007). Sodium-hydrogen exchangers (NHEs), proteins involved in regulating intracellular pH through sodium and hydrogen ion exchange, have shown overexpression of NHE1 in gastric cancer cells. NHE1 promotes cell proliferation in gastric cancer by modulating the G1/S and G2/M cell cycle transitions, while upregulating positive cell cycle regulators like cyclin D1 and cyclin B1 in gastric cancer cells. Furthermore, NHE1 stimulates the proliferation, invasion, and migration of gastric cancer cells by influencing the expression of epithelial-mesenchymal transition (EMT) proteins (Xie et al., 2017a). Sodium-potassium ATPase (Na^+^/K^+^ ATPase), an ion pump responsible for maintaining cellular ion balance, has been investigated, revealing that silencing ATP1B3 leads to reduced expression of phosphatidylinositol 3-kinase (PI3K), protein kinase B (AKT), and phosphorylated AKT (p-AKT), ultimately inhibiting the proliferation and migration of gastric cancer cells (Li et al., 2017). Sodium-dependent glucose transporters (SGLTs), which utilize sodium gradients to facilitate glucose transportation into cells, have been found to exhibit high expression of the SGLT1 gene in gastric cancer, correlating with a poorer prognosis for gastric cancer patients. SGLT1 promotes gastric cancer cell proliferation and glucose metabolism, exerting carcinogenic effects in gastric cancer (Shi et al., 2022). Recent investigations have revealed alterations in sodium ion channels during the onset and progression of gastric cancer, particularly highlighting significantly higher expression of the Nav1.7 channel in gastric cancer tissues compared to normal gastric tissues. Abnormal expression of the Nav1.7 sodium ion channel in gastric cancer cells closely aligns with malignant severity (Xia et al., 2016).

The aforementioned research findings highlight the involvement of sodium ions, fundamental for maintaining homeostasis, in regulating the characteristics of tumor biology. Further investigation into the relationship between sodium ion levels and the diagnosis, progression, and prognosis of gastric cancer is warranted to offer insights for therapeutic interventions based on this fundamental examination.

### 2.2 Iron

Iron (Fe2^+^,Fe3^+^), an indispensable element found in numerous enzymes such as hemoglobin, myoglobin, and cytochromes, plays a vital role as a catalytic subunit, facilitating electron transfer and mediating redox reactions (Philpott, 2012). While its significance is undeniable, excessive iron poses a threat to organisms due to its potent oxidizing ability, leading to the generation of toxic oxygen species through the Fenton reaction. (Koppenol and Hider, 2019).

Rahman et al. (Rahman et al., 2020) conducted an investigation exploring the association between iron and gastric cancer, revealing that iron levels below 30 mg/L increase the susceptibility to *H. pylori* infection, consequently influencing the incidence of gastric cancer (Lahner et al., 2018). Moreover, a substantial reduction in gastric acid secretion occurs when iron deficiency reaches 35% of the recommended amount. This reduction exacerbates the progression of gastric cancer, resulting in shortened survival rates for patients diagnosed with the disease (Rahman et al., 2020). The research by Rahman (Rahman et al., 2020) and Noto (Alizadeh and Raufman, 2022) iron deficiency contributes to gastric carcinogenesis through the induction of gastric inflammation, thereby promoting disease progression. However, some scholars have also identified a significant direct correlation between increased heme iron intake (>3.14 mg/day) and the risk of distal gastric cancer (Epplein et al., 2014). Hence, the role of iron in the onset and advancement of gastric cancer holds crucial significance, with its intertwined relationship with *H. pylori*, albeit the precise underlying mechanisms necessitating further elucidation. Studies indicate that Fe^2+^ creates a tumor-promoting microenvironment that facilitates carcinogenesis, including gastric cancer (Gu et al., 2022). Liang et al. further demonstrated the involvement of iron ions, elucidating one of the mechanisms through which iron ions foster gastric cancer development by generating reactive oxygen species (ROS), which contribute to DNA damage and the activation of carcinogenic signaling pathways (Liang et al., 2016).

**FIGURE 3:**
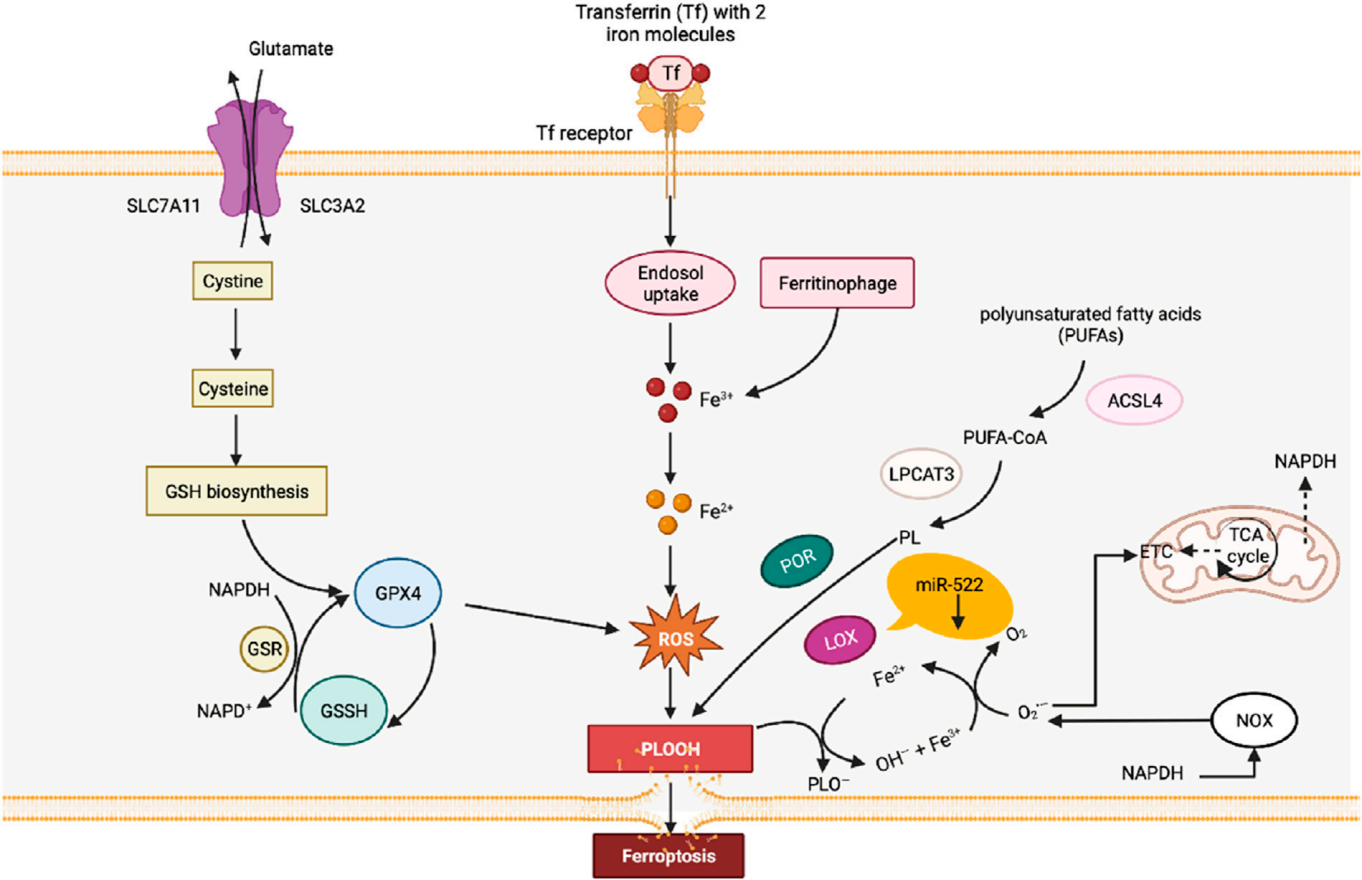
Diagram of the relationship between lipid peroxidation and ferroptosis. The process of lipid peroxidation in cells is mainly catalyzed by the lipid peroxidation process catalyzed by fatty acid enzymes and the Fenton reaction induced by free iron ions. At the same time, the clearance of lipid peroxidation in cells mainly relies on the action of glutathione peroxidase 4 (GPX4).

Cellular iron death represents a form of cell death induced by the accumulation of iron-dependent lipid peroxides. Primarily attributed to iron overload and the buildup of reactive oxygen species (ROS) (Figure 3), this phenomenon extensively affects mitochondrial utilization, breakdown, and synthesis pathways. During iron death, mitochondria display distinctive characteristics, including heightened membrane density, reduced volume, diminished or absent cristae, and ruptured outer membranes in comparison to normal mitochondria (Jiang et al., 2021). Remarkably, gastric cancer cells use diverse pathways to resist iron death. For instance, cancer-associated fibroblasts (CAFs) impede iron death by releasing extracellular vesicle miR-522, which targets ALOX15 and impedes lipid ROS accumulation (Zhang et al., 2020). Additionally, the Wnt/β-catenin signaling pathway in gastric cancer cells reinforces resistance to iron death by targeting GPX4 (Wang et al., 2022). The activation of the MAT2A-ACSL3 pathway in gastric cancer cells also contributes to cellular resistance against iron death (Ma et al., 2022).

Emerging investigations have elucidated the participation of numerous functionally significant proteins in the modulation of ferroptosis during the developmental stages of gastric cancer. Notably, cysteine dioxygenase 1 (CDO1) assumes a pivotal role in the mechanism of iron-dependent cell death provoked by Erastin in gastric cancer cells. Suppression of CDO1 impedes Erastin-triggered ferroptosis in gastric cancer cells by augmenting intracellular levels of glutathione and the expression of GPX4, thereby abating the generation of reactive oxygen species and lipid peroxidation. Consequently, the process of iron-dependent cell death in gastric cancer cells is impeded (Zhao et al., 2020). Akin to CDO1, stearoyl-CoA desaturase 1 (SCD1), an enzyme associated with the endoplasmic reticulum that is significantly upregulated in gastric cancer tissues, is implicated in the conversion of saturated fatty acids to monounsaturated fatty acids. Upregulation of SCD1 correlates with the growth of gastric cancer cells and the inhibition of ferroptosis, underscoring its potential as a prognostic marker for gastric cancer (Wang et al., 2020). Furthermore, microRNAs (miRNAs), a class of non-coding small RNA molecules composed of 18–24 nucleotides, have been implicated in the dysregulation of ferroptosis in gastric cancer cells through their modulation of gene expression via interactions with the 3′untranslated region (3′UTR) of target genes (Lu et al., 2022). For instance, the upregulation of miR103a3p, an oncogenic miRNA, in gastric cancer is associated with an unfavorable prognosis in patients (Hu et al., 2018). Niu et al. discovered that emodin-8-glucoside (EG), a compound derived from Rheum palmatum, induces ferroptosis in gastric cancer cells, suppressing their proliferation and metastasis by alleviating the inhibitory effect of miR103a3p on phospho-activated glutaminase 2 (GLS2) (Niu et al., 2019). GLS2, a glutaminase modulator induced by p53, converts glutamine to glutamate for synthesizing glutathione (Kang et al., 2019), and miR103a3p regulates ferroptosis in gastric cancer cells by altering intracellular glutathione levels. Furthermore, Mao et al. discovered that the local anesthetic levobupivacaine induces ferroptosis in gastric cancer cells through the miR4893p/SLC7A11 axis, thereby inhibiting gastric cancer cell growth (Mao et al., 2021). In terms of signaling pathways in gastric cancer cells, iron ions are involved in the regulation of AKT/mTOR (Xu et al., 2020), ERK (Geng and Wu, 2022), NF-κB (Yao et al., 2021), STAT3 (Zuo et al., 2018), Wnt (Wang et al., 2022), and other pathways, indicating their significant impact on the occurrence and progression of gastric cancer through activation or inhibition of these pathways. Regarding the mechanisms of iron entry into gastric cancer cells, transferrin receptor (TfR) expression does not differ significantly from normal gastric tissue (Dong et al., 2012), but recent studies suggest that TfR could be a novel prognostic marker and therapeutic target (Yue et al., 2012).

In the context of investigating gastric cancer drug resistance, research has demonstrated that the upregulation of the KEAP1/NRF2 signaling pathway significantly diminishes the sensitivity of gastric cancer cells to fluorouracil (5-FU) and oxaliplatin (Zhou et al., 2023). Moreover, the KEAP1/NRF2 pathway exhibits a close association with ferroptosis. For instance, the knockout of latent transforming growth factor-beta binding protein 2 (LTBP2) augments ferroptosis in gastric cancer cells via the KEAP1/NRF2 pathway (Wang et al., 2022), while Xiaojian Decoction, a traditional Chinese medicine, can alleviate gastric mucosal injury by suppressing ferroptosis through the activation of the Keap1/Nrf2 signaling pathway in normal gastric tissue (Chen et al., 2022). Additionally, downregulation of the negative regulatory axis involving stat3 in ferroptosis can reduce the resistance of typical chemotherapeutic drugs such as 5-FU (Ouyang et al., 2022). Furthermore, ATF3 has been found to induce ferroptosis and enhance sensitivity to cisplatin in GC cells by blocking Nrf2/Keap1/xCT signaling (Fu et al., 2021).

In the treatment of gastric cancer, resistance to cisplatin and paclitaxel has become increasingly severe in GC patients (Zhai et al., 2019), and ferroptosis inducers may help overcome this resistance. Studies have indicated that blocking lipid reactive oxygen species (ROS) mediated by cancer-associated fibroblast exosomes leads to increased levels of ferroptosis in cancer cells, thus enhancing their sensitivity to chemotherapy (Zhang et al., 2020). Another potential therapeutic target for gastric cancer treatment is the GCN2-eIF2α-ATF4-xCT pathway, which is activated by ROS and constitutes a signaling cascade that amplifies resistance to cisplatin via the induction of mitochondrial dysfunction (Wang et al., 2016). Moreover, modulation of ROS levels represents a novel therapeutic strategy since ROS can disrupt cellular oxidative environments and induce cell death. Peroxiredoxin 2, an antioxidant enzyme, significantly sensitizes AGS and SNU-1 cells to cisplatin treatment by regulating ROS levels (Dharmaraja, 2017). Regulating ferroptosis may thus serve as a practical strategy for targeting drug-resistant tumor cells (Zhang et al., 2021) given that chronic and excessive ROS levels contribute to drug resistance (Xu et al., 2020).

### 2.3 Copper

Copper (Cu^2+^) represents an essential elemental component ubiquitous in nearly all living organisms and assumes the role of a cofactor for crucial metabolic enzymes that orchestrate various physiological processes. However, maintaining precise copper levels proves vital for sustaining normal biochemical reactions (Scheiber et al., 2013). Extensive research has focused on unraveling the impact of copper on cancer progression due to its potential involvement in the activation of cell proliferation-related signaling pathways. Notably, cancer cells, including those in gastric tissue, generally exhibit heightened copper requirements in comparison to their normal cell counterparts (Denoyer et al., 2015). Furthermore, serum copper levels have demonstrated correlations with tumor staging and disease progression across multiple cancer types, including gastrointestinal malignancies (Diez et al., 1989; Gupta et al., 1993).

Copper emerges as a pivotal factor in cellular signal transduction, contributing to the development and progression of cancers such as gastric cancer by stimulating cell proliferation, angiogenesis, and metastasis. Experimental evidence involving mice indicates that direct administration of copper promotes the growth of tumors such as pancreatic, lung, and breast cancer (Ishida et al., 2013; Skrajnowska et al., 2013; Brady et al., 2014). Mechanistically, copper can amplify the generation of reactive oxygen species (ROS), which closely associates with cellular malignant transformation. Additionally, the copper transporter CTR1-dependent import mechanism triggers the mitogen-activated protein kinase (MAPK) signaling cascade (Turski et al., 2012). Copper forms direct high-affinity bonds with MEK1, thereby fostering tumor growth via the activation of downstream ERK1/2 phosphorylation (Brady et al., 2017). Furthermore, copper acts as a fundamental regulator of the autophagy kinase ULK1/2, thereby facilitating carcinogenesis (Tsang et al., 2020). The promotion of vascular growth constitutes a critical aspect of tumor progression, and investigations by McAuslan (Mcauslan and Reilly, 1980) have established the ability of copper to facilitate blood vessel formation. Silencing the expression of CTR1 in endothelial cells impedes copper entry, consequently reducing cell migration and ultimately attenuating angiogenesis (Narayanan et al., 2013). Copper is also closely associated with cancer metastasis, as it can activate metastasis-related enzymes and signaling cascades. Copper enzyme LOX is involved in tumor cell invasion (Erler et al., 2009) and metastasis and has been identified as an important factor in breast cancer metastasis (Cox et al., 2015). Additionally, copper impacts PD-L1, an immune checkpoint inhibitor implicated in cancer immune evasion. Studies have highlighted that depleting copper facilitates the degradation of PD-L1, inhibiting tumor growth and enhancing survival rates in animal models (Voli et al., 2020).

Regarding the resistance of cancer cells, including gastric cancer cells, to platinum-based chemotherapy, ongoing research suggests that the interaction between the copper ion transporter ATP7B and cisplatin disrupts copper homeostasis, thereby augmenting resistance to platinum-based drugs (Leonhardt et al., 2009).

**FIGURE 4:**
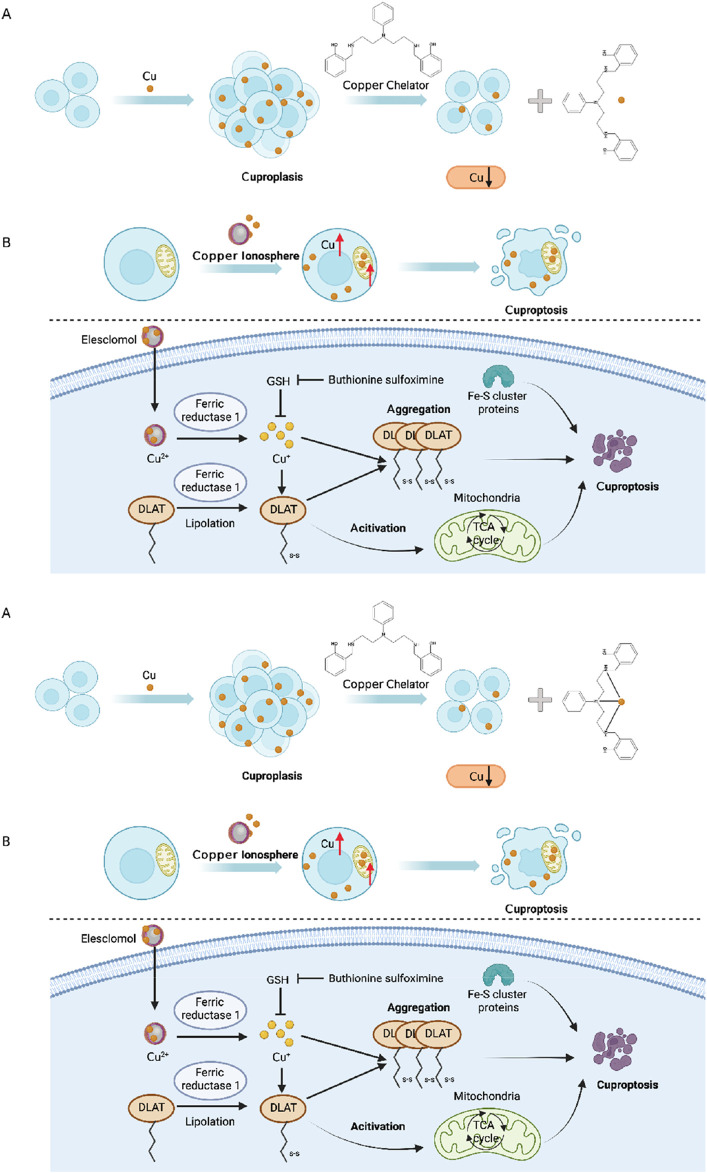
**(A)** Cu chelating agents reduce Cu bioavailability; **(B)** Cu ionophores deliver Cu into cells to increase intracellular Cu levels. Excessive Cu induces oligomerization of Lipoyl Acyltransferase (DLAT). The oligomerization of DLAT leads to cell toxicity and induces cell death. At the same time, FDX1 reduces Cu2^+^ to more toxic Cu^+^, ultimately leading to inactivation of Fe-S proteins. Together, they induce protein toxicity stress, ultimately resulting in cell death.

In the realm of cancer treatment, the disruption of copper homeostasis in tumor cells, coupled with the substantial role of copper in promoting cancer progression, has spurred the development of several copper coordination compounds for anticancer therapy. These compounds can be broadly categorized into two major groups: copper chelators that diminish copper bioavailability (Figure 4A) and copper ion carriers that facilitate copper delivery into cells, elevating intracellular copper levels (Figure 4B). Prominent copper chelators employed in current research encompass TTM, trientine, and d-penicillamine, among others, with their anticancer activities substantiated through diverse animal models and clinical trials (Zagzag et al., 1990; Yoshii et al., 2001). Nevertheless, research targeting gastric cancer in particular remains scarce, thus providing an avenue worthy of investigation. As for copper ion carrier drugs, elesclomol, bis(thiosemicarbazone) analogs, and disulfiram (DSF) have exhibited heightened intracellular copper levels and displayed anticancer activity. The cytotoxic effects of ion carriers can be attributed to their capacity to intensify ROS production and inhibit proteasomes (Denoyer et al., 2016). Specifically, the combination of DSF and copper (DSF/Cu) demonstrates promise in overcoming drug resistance during gastric cancer chemotherapy. DSF/Cu sensitizes tumor cells to cisplatin by targeting aldehyde dehydrogenase (ALDH+) (Macdonagh et al., 2017), impedes ATP hydrolysis to prevent drug efflux (Shukla et al., 2004), enhances the sensitivity of tumor cells to temozolomide by inhibiting proteasomes (Lun et al., 2016), and reduces nuclear factor κB (NF-kB) activity to delay IkB degradation, thereby increasing cell sensitivity to gemcitabine (Guo et al., 2010). DSF/Cu also reverses resistance to tamoxifen by amplifying the expression and phosphorylation of c-Jun NH2-terminal kinase (JNK) (Xu et al., 2011). These findings offer novel insights into the diagnosis and treatment of gastric cancer through chemotherapy. Elesclomol, a pioneering copper ion carrier, effectively transports copper to mitochondria, culminating in escalated oxidative stress and cell death. Beyond the cell, elesclomol forms a stable 1:1 complex with Cu(II) (Wu et al., 2011). Subsequently, elesclomol freely shuttles between extracellular and intracellular environments, facilitating the delivery of copper ions into cells. Distinguishing itself from other copper ion carriers like DSF, elesclomol selectively enhances copper levels in mitochondria. At equivalent concentrations, elesclomol induces a significantly greater elevation of intracellular copper ions compared to DSF (Nagai et al., 2012). Moreover, treatment with elesclomol has been shown to degrade copper efflux protein ATPase 1 (ATP7A) in colon cancer cells, responsible for mediating copper efflux (Fukai et al., 2018). The degradation of ATP7A by elesclomol further enriches copper ions within cancer cell mitochondria (Gao et al., 2021). Elesclomol has demonstrated the potential to enhance the therapeutic efficacy of paclitaxel in patients with refractory solid tumors (O'Day et al., 2013). Thus, exploring the application of Elesclomol in combination with copper for cancer treatment, particularly in gastric cancer, represents a promising avenue for future research.

### 2.4 Potassium

Potassium ions (K^+^) are vital cellular ions that play crucial roles in maintaining intracellular and extracellular fluid ion balance, as well as regulating muscle and nervous system function and cellular signaling processes (Palmer and Clegg, 2016). In the context of gastric cancer, potassium ions have been identified to have a significant impact. Existing research supports the notion that higher levels of potassium intake, ranging from 2.5 to 2.8 g/d, are associated with a reduced risk of gastric cancer (Choi et al., 2022; Tran et al., 2022). In terms of signaling pathways related to potassium ions, monovalent cations like K^+^ primarily modulate membrane potential and indirectly influence other ion signaling pathways, in contrast to divalent cations such as Ca^2+^ and Mn^2+^ which act as second messengers (Stone et al., 2016).

Numerous potassium channels have been identified as important contributors to the development and progression of gastric cancer (refer to Table 1) (Anderson et al., 2019). In the context of drug resistance, Kv1.5 expression is widespread among various gastric cancer cells, including SGC7901. Upregulation of Kv1.5 leads to increased K (+) current density in gastric cancer cells, resulting in enhanced sensitivity to different chemotherapeutic drugs. Conversely, downregulation of Kv1.5 promotes drug resistance in gastric cancer cells (Han et al., 2007). *In vivo* studies have shown that knockout of Kir2.2 suppresses tumor cell proliferation and metastasis by upregulating the tumor suppressor p27, increasing the accumulation of ROS, and downregulating cyclin A, cdc2, and E2F1. Among the Kv channel family, Kv4.1, Kv7.1 (KCNQ1), and Kv1.5 have been identified as participants in promoting the proliferation and progression of gastric cancer cells (Lee et al., 2000; Arcangeli et al., 2009; Kim et al., 2010). Furthermore, downregulation of the KCNQ1 subunit KCNE2 in gastric cancer inhibits cell proliferation and tumor occurrence in the stomach (Yanglin et al., 2007). Additionally, although Kv11.1 is undetectable in normal gastric tissue, it is detected in GC tissue (Ding et al., 2010) and has been shown to promote GC proliferation and tumor occurrence both *in vitro* and *in vivo*, regulating the secretion of vascular endothelial growth factor 1 (VEGF-1) through the AKT-dependent pathway (Shao et al., 2008; Crociani et al., 2014). Moreover, Kv11.1 has been identified as crucial for inducing apoptosis in gastric cancer cells mediated by cisplatin, suggesting its potential as a novel target for cisplatin chemotherapy (Zhang et al., 2012).

**TABLE 1:** Gene expression of K+ channel on gastric cancer.

Gene name	Cell localization	Physiological function	Pathophysiological conditions caused by dysfunction in GC	Ref.
KCNQ1(K_V_7.1)	Apical	KCNQ1 participates in K^+^ recycling and stimulates gastric acid secretion; Pumps K^+^ into the lumen	KCNQ1 is implicated in GC progression	Bielanska et al. (2009), Zhang et al. (2013)
KCNA5/Kv1.5	Apical	Pumps K^+^ into the lumen	Expression upregulated; silencing in GC cells inhibits proliferation; alters drug resistance	Lan et al. (2005), Han et al. (2007), Arcangeli et al. (2009a)
KCND1/Kv4.1	Apical	Pumps K^+^ into the lumen	Expression upregulated	Kim et al. (2010)
KCNE2/MiRP1	Apical	KCNE2 participates in K^+^ recycling and stimulates gastric acid secretion; Pumps K^+^ into the lumen	Expression downregulated; deficiency promotes tumor progression; knockout mice develop gastritis cystic profundal and neoplasia, pyloric polyadenomas; invasive adenocarcinomas; upregulation of cyclin D1; downregulated in gastric cancer tissues and cell lines; overexpression in cell lines suppressed growth in soft agar and mouse tumor xenografts	Yanglin et al. (2007), Roepke et al. (2010), Kuwahara et al. (2013), Abbott and Roepke (2016), Li et al. (2016)
NKCC1/SLC12A2	Basolateral	NKCC1/SLC12A2 known as Na^+^-K^+^-2Cl^−^ cotransporter pumps Na^+^, K^+^, and 2Cl^−^ into parietal cells	NKCC1 promotes proliferation, invasion and migration in human GC cells via activation of the MAPK-JNK/EMT signaling pathway	Wang et al. (2021)
KCNH2/hERG1/Kv11.1	Apical	Pumps K^+^ into the lumen	Expression upregulated; stimulates angiogenesis by promoting VEGF-A secretion via AKT-dependent regulation of HIF1; promotes GC cell proliferation and progression with positive in 69% of gastric cancers; associated with poor patient prognosis	Arcangeli et al. (2009b), Ding et al. (2010), Crociani et al. (2014), Lang and Stournaras (2014), Arcangeli and Becchetti (2017)

^a^ GC: gastric cancer.

Potassium ions play a crucial role as essential metal ions in the human body. Adequate intake of potassium (K) has been shown to have a positive impact on the prevention of gastric cancer. Extensive research has focused on studying the structure and function of potassium ion channels, revealing significant alterations in certain channels during the occurrence and progression of gastric cancer. These channels hold promise as potential targets for the treatment of gastric cancer.

### 2.5 Zinc

Zinc (Zn^2+^) is a constituent element of the human body, with a total content ranging from 2 to 3 g. Daily exchange of approximately 0.1% occurs, and the majority (around 90%) is localized in muscles and bones. The presence of zinc is crucial for the activity or tertiary structure formation of over 10% of human genes, including transcription factors, receptors, kinases, ligases, and enzymes. These proteins, encompassing more than three thousand in number, require zinc binding to facilitate their catalytic functions (Elitt et al., 2019).

In the context of gastric cancer, Yuan’s (Yuan et al., 2016) research findings propose a potential link between alterations in the Cd/Zn ratio and the development of gastric cancer. These alterations may contribute to elevated error rates in DNA replication and ineffective DNA repair, potentially promoting carcinogenesis. Additionally, Namikawa et al. (2021) discovered a noteworthy correlation between low levels of zinc and gastric cancer, with a significant portion (68.8%) of gastric cancer patients exhibiting serum zinc deficiency (<80 μg/Dl). Notably, zinc deficiency has been associated with unfavorable prognosis in gastric cancer patients. A pioneering study conducted in Korea demonstrated that sufficient zinc intake significantly prolongs the survival of gastric cancer patients. The insufficiency of zinc intake can result in reduced immune function, compromised DNA damage response, and repair capabilities (Kwak et al., 2022). While previous research suggests the involvement of zinc in the regulation of pathways such as Wnt/β-catenin, NF-κB, and PI3K/Akt, its specific role in gastric cancer cells remains unclear (Didonato et al., 2012; Tian et al., 2020; Liang et al., 2022).

Due to its considerable cytotoxicity at high concentrations, cellular zinc levels are stringently regulated. Zinc does not pass freely through the cell membrane; instead, it is regulated by various zinc permeation channels and transport proteins responsible for cellular uptake and efflux (Levaot and Hershfinkel, 2018). These transporters include influx and efflux transporters, while zinc can also traverse the cell membrane through other ion channels. Notably, Zn^2+^ can activate certain voltage-gated Ca^2+^ ion channels permeable to zinc (Inoue et al., 2015). Disturbances in the ZnT/SLC30A and ZIP/SLC39A families, which constitute major groups of Zn^2+^ transporters, have been observed in gastrointestinal cancer (Fukada and Kambe, 2011). In gastric cancer, the expression levels of ZIP1, 2, 4, 6, 7, 8, 9, 11, 12, 13, and 14 zinc transport proteins are notably elevated. Specifically, ZIP7, 11, and 14 expression levels correlate positively with patient survival (Anderson et al., 2019). However, the mechanisms underlying dysregulation of zinc homeostasis and the impact of zinc transport protein expression in cancer remain unclear. Existing research only provides insights into the tissue-specific roles of zinc in different types of cancers. For example, zinc transport proteins act as tumor suppressors in prostate cancer but as oncogenes in breast cancer (Bhutia et al., 2016; Takatani-Nakase, 2018). The specific role of zinc in gastrointestinal cancers, including gastric cancer, is still inadequately understood. Changes in intracellular zinc ion concentrations can serve as second messengers for external signals, including the activation of pathways such as mitogen-activated protein kinase (MAPK), extracellular signal-regulated kinase (ERK), and c-Jun N-terminal kinase (JNK). These pathways play crucial roles in protein phosphorylation and the regulation of fundamental cellular processes such as proliferation, differentiation, and apoptosis (Bafaro et al., 2017; Levaot and Hershfinkel, 2018). Consequently, zinc is considered a pivotal signaling molecule in both normal cellular functions and pathological conditions, including cancer. Nonetheless, the precise mechanisms by which zinc signaling is transduced from zinc transporters to downstream signaling pathways remain elusive.

**FIGURE 5:**
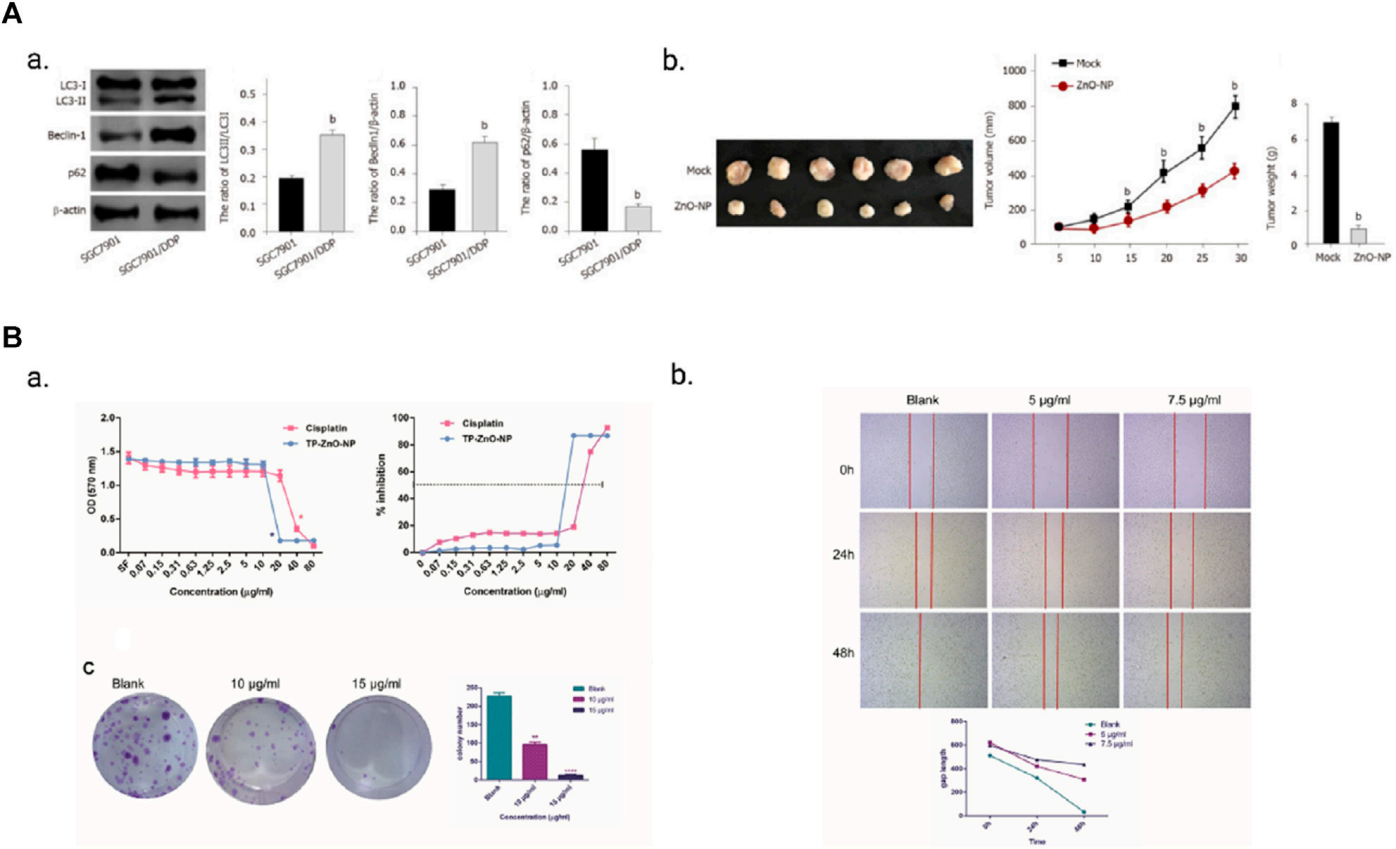
**(A)** ZnO-NP can reduce tumor growth of chemotherapy-resistant GC cells *in vivo*. Adopted from MIAO Y H, MAO L P, CAI X J, et al. Zinc oxide nanoparticles reduce the chemoresistance of gastric cancer by inhibiting autophagy. World J Gastroenterol, 2021, 27 (25): 3,851-62; **(B)** TP-ZnO-NP has significant anti-proliferation and anticancer activity on gastric cancer cells. TP-ZnO-NPs demonstrated better inhibition on the proliferation and colony formation of gastric cancer cells compared with cisplatin. Adopted from BOZGEYIK I, EGE M, TEMIZ E, et al. Novel zinc oxide nanoparticles of Teucrium polium suppress the malignant progression of gastric cancer cells through modulating apoptotic signaling pathways and epithelial to mesenchymal transition. Gene, 2023, 853: 147091.

The resistance of gastric cancer cells to drugs is closely associated with the presence of zinc. Several zinc finger proteins have been identified as contributors to drug resistance in gastric cancer cells. ZFP64 overexpression, for instance, is associated with invasive phenotypes and resistance to nab-paclitaxel. The ZFP64/GAL-1 axis promotes the therapeutic effect of nab-paclitaxel and counteracts the immunosuppressive microenvironment in gastric cancer (Zhu et al., 2022). Similarly, overexpression of zinc finger protein GLI1 induces drug resistance in gastric cancer cells by binding to the AKT-mTOR pathway (Yao et al., 2019). The regulatory role of MALAT1 in oxaliplatin (OXA) resistance in gastric cancer cells is mediated by its interaction with ZFP91 through miR-22-3p sponging (Zhang et al., 2020). Additionally, zinc finger protein 139 (ZNF139) inhibits the expression of multidrug resistance (MDR)-related genes in gastric cancer cells, resulting in multidrug resistance (Tan et al., 2018). Notably, zinc, when employed as a nanomaterial, demonstrates advantageous effects in overcoming drug resistance in gastric cancer cells. For instance, zinc oxide nanoparticles (ZnO-NP) alleviate drug resistance in gastric cancer cells by inhibiting autophagy (Miao et al., 2021). Noteworthy findings reveal that SGC7901/DDP cells, which are resistant to DDP, exhibit increased autophagy levels compared to SGC7901 cells. Treatment with ZnO-NP (5 μg/mL) leads to decreased tumor growth in SGC7901/DDP cells in nude mice, thereby indicating a reduction in tumor growth of chemotherapy-resistant gastric cancer cells (Figure 5A). Furthermore, TP-ZnO-NP, another novel nanomaterial, exhibits significant anti-proliferative and anti-cancer activity against gastric cancer cells (Bozgeyik et al., 2023). TP-ZnO-NP effectively inhibits the viability of gastric cancer cells in a dose-dependent manner after a 24-h incubation period. Colony formation assays further support the interference of TP-ZnO-NP with the colony-forming ability of HCG-27 cells in a dose-dependent manner. Moreover, TP-ZnO-NP significantly suppresses the migratory capacity of HCG-27 gastric cancer cells in a time- and dose-dependent manner (Figure 5B).

Limited research has been conducted regarding the development of drugs that specifically target various zinc transporters for the treatment of gastric cancer. However, immediate therapeutic effects have been observed through zinc supplementation in diseases related to zinc deficiency, including cancer. The efficacy of zinc supplementation has been demonstrated in the treatment of inflammatory bowel disease and may be beneficial for zinc-deficient cancers (Franklin et al., 2014). Additionally, zinc finger proteins may play a crucial role in enhancing the adjunctive chemotherapy process for gastric cancer (Zhu et al., 2022).

### 2.6 Calcium

The role of calcium (Ca^2+^) is multifaceted in gastric cancer, acting as a ubiquitous second messenger and a signaling molecule involved in various cellular processes such as cell cycle control, apoptosis, and migration. Calcium ions exhibit the highest concentration gradient among metal ions in the body, with uneven subcellular distribution. Most intracellular calcium is stored in the extracellular space and the endoplasmic reticulum, with concentrations ranging from 0.3 to 2 mM (Prakriya, 2020). In the event of endoplasmic reticulum stress, calcium is released into the cytoplasm, activating calcium-dependent proteases in proximity to the endoplasmic reticulum. These proteases can impact Caspase-12, leading to its activation and subsequent release into the cytoplasm, thereby inducing apoptosis. Additionally, calcium can induce apoptosis by activating calcium/calmodulin-dependent protein phosphatase, which triggers the dephosphorylation of the pro-apoptotic protein Bad, consequently resulting in the release of cytochrome C (Brenner and Mak, 2009). However, gastric cancer cells exhibit resistance to calcium-induced apoptosis via the mitochondrial pathway. Mechanisms underlying this resistance involve the overexpression of Cell Retinoic Acid Binding Protein 2 (CRABP2), which promotes the binding of BAX and PARKIN in gastric cancer cells, thereby facilitating ubiquitin-mediated BAX degradation and weakening of mitochondrial apoptosis (Tang et al., 2022). Moreover, Ajuba overexpression in gastric cancer regulates mitochondrial membrane potential through the YAP/Bcl-xL/GLUT1 pathway, offering resistance against apoptosis (Li et al., 2019). Additionally, MUC20 in gastric cancer cells plays a significant role in maintaining mitochondrial homeostasis (Fu et al., 2022).

Calcium ions demonstrate contradictory roles in gastric cancer. Some studies suggest that calcium intake may have a promoting effect on gastric cancer. Xie et al. (2017b) found that calcium enhances the expression and function of the calcium-sensing receptor (CaSR), potentially promoting the effects of GC. Similarly, Wang et al. (2020a) demonstrated that elevated calcium levels of 0.1–2 mM in gastric cancer cells promote tumor growth, proliferation, and metastasis through the AKT/β-catenin pathway. Conversely, other studies indicate that calcium intake can reduce the risk of gastric cancer. Dai et al. (2013) observed that a daily calcium intake of over 600 mg reduces the risk of gastric adenocarcinoma, while Shah et al. (2020) reported that calcium intake exceeding 881.3 mg/day can lower cancer-related mortality. Though the above research might appear contradictory, Dai, Shah and their teams conducted epidemiological studies on a large population while Xie, Wang and their groups explored the effects of calcium ions on gastric cancer *in vitro*. Given that the conversion and distribution of calcium ions within the human body are extremely complex, this implies that the expression of calcium ingested from diet or supplements differs from that of calcium generated through chemical reactions surrounding gastric cancer cells. Patergnani et al. (2020) altered Ca^2+^ signaling can disrupt calcium dynamics, affecting various aspects of cellular function. Excessive Ca^2+^ overload can lead to mitochondrial swelling, rupture, and cell death, contributing to widespread apoptosis and ultimately carcinogenesis. Proliferation and cell growth, which may lead to various types of cancer including gastric cancer, are among the cellular functions affected. Furthermore, the accumulation of calcium ions (Ca^2+^) in the cytoplasm can cause cellular calcium overload, inducing cell death (Bai et al., 2022).

While the precise role of calcium ions in gastric cancer development requires further elucidation, several mechanisms have been proposed to explain their potential involvement. TRP channels, consisting of transmembrane proteins, regulate ion distribution between cells by forming gated pores as homotetramers or heterotetramers. Notably, six TRP channels (TRPC6, TRPM2, TRPM5, TRPM7, TRPV4, and TRPV6) have been identified as crucial players in the growth and survival of gastric cancer (Table 2) (Sterea et al., 2019). Cai et al. (2009) an upregulation of TRPC6 expression in human gastric cancer tissue and revealed that inhibiting TRPC6 significantly halts the cell cycle at G2/M phase, resulting in a notable reduction in cell growth. TRPC6 also plays a critical role in the epithelial-to-mesenchymal transition (EMT) of gastric cancer through regulation of the Ras/Raf/ERK1/2 signaling pathway (Ge et al., 2018). Almasi et al. (2019) confirmed the significance of TRPM2 in gastric cancer by downregulating its expression using targeted TRPM2 shRNA in gastric cancer cell lines, leading to inhibited invasion and reduced cell survival rates. These findings were further supported by an *in vivo* model using SCID mice, where the loss of TRPM2 resulted in decreased tumor growth, primarily mediated by the jnk-dependent and mTOR-independent autophagy pathway (Almasi et al., 2018). TRPM2 expression levels showed a negative correlation with patient survival rates (Almasi et al., 2019). Similarly, other studies have found a correlation between high expression of TRPM5 and shorter survival periods in gastric cancer patients (Maeda et al., 2017). identified an upregulation of TRPM7 in many gastric cancer cell lines and demonstrated that inhibiting TRPM7 significantly reduced cell proliferation and increased apoptosis in gastric cancer cells Kim et al. (2008). TRPV4 also plays a significant role in gastric cancer, with studies confirming its upregulation in gastric cancer cells. Activation of TRPV4 induces a substantial increase in cytoplasmic Ca^2+^ levels through a large outward rectifying current (Xie et al., 2017b). The functional role of TRPV4 in gastric cancer cells is achieved via the activation of G protein-coupled receptors (GPCRs) (Tang et al., 2019). Hediger (Peng et al., 2000) found an upregulation of TRPV6 expression in gastric cancer cells, and subsequently, demonstrated that capsaicin/TRPV6 can induce cell death in gastric cancer cells through the Ca^2+^/p53/JNK pathway Chow et al. (2007).

**TABLE 2:** Therapeutical agents and changes in the expression or activity of some Ca^2+^ channels and pumps in gastric cancer.

Channel or pump		Changes in cancer			Activator	Inhibitors	Ref.
Function	Name	mRNA	Protein	Activity			
Store Ca^2+^ channel	IP_3_R3	upregulated	upregulated	—	—	Heparin, polyvinyl sulphate	O'Rourke and Feinstein (1990), Takahashi et al. (1994), Al et al. (2003)
Voltage-gated channels	Ca_V_3.1 (T-type α_1G_)	downregulated	—	Expression downregulated by promoter hypermethylation	—	—	Lafrenie et al. (2017)
	CACNA2D3	downregulated	downregulated	Expression downregulated by promoter hypermethylation	—	—	Lastraioli et al. (2015)
Transient receptor potential channels	TRPC6	upregulated	upregulated	—	—	Pyrazolo [1,5-a]pyrimidine	Cai et al. (2009), Brabletz et al. (2018), Ding et al. (2018), Ge et al. (2018)
	TRPM2	upregulated	upregulated	Suppression reduced proliferation of gastric cancer cells, increased autophagy and sensitized cells to paxlitaxel and doxorubicin	—	*N*-(p-amylcinnamoyl)anthranilic acid, 2-aminoethoxydiphenyl borate, clotrimazole	Jiang et al. (2010), Xia et al. (2017), Almasi et al. (2018), Lin et al. (2018)
	TRPM7	High-expression	High-expression	Mg is required for GC survival; Inhibitors induced cell death		Quercetin, ginsenoside Rd, ginsenoside Rg3	Kim et al. (2011); Kim (2013), Kim et al. (2014), Litan and Langhans (2015)
	TRPV1	downregulated	downregulated	—	Evodiamine	—	Gao et al. (2020), Liu et al. (2022)

“—”: no data or no available pharmacological agents; TRPC, transient receptor potential canonical.

In the realm of gastric cancer research, the involvement of calcium channels and calcium-binding proteins has garnered significant attention. Notably, the histidine-rich calcium-binding protein (HRC), a calcium-binding protein, has been observed to modulate the Raf/MEK/ERK pathway through calcium (Ca) and calmodulin (CaM) signaling, thereby impacting the epithelial-mesenchymal transition (EMT) in gastric cancer (GC) cells (Wang et al., 2022). Another calcium-binding protein, S100A14, exhibits the ability to upregulate the expression of E-cadherin and PGII, promoting differentiation in GC cells and inhibiting metastasis (Zhu et al., 2017). Similarly, the calcium-binding protein/Siah-1 interacting protein (CacyBP/SIP) has been found to inhibit cell growth and invasion in gastric cancer cells by activating β-catenin expression and Tcf/LEF transcription (Ning et al., 2007).

Furthermore, the influence of calcium ions extends to the acquisition of drug resistance in gastric cancer cells. Studies indicate that overexpression of MUC20v2 in GC cells leads to chemoresistance against cisplatin (CDDP) and paclitaxel (PTX), with upregulated pathways related to intracellular calcium regulation. This finding supports the notion that forced expression of MUC20v2 in the cytoplasm of GC cells contributes to the maintenance of mitochondrial calcium homeostasis and mitochondrial membrane potential (MMP), thereby conferring chemoresistance (Fu et al., 2022). Additionally, calcium ion channels, such as TRPA1 and TRPC5, have been implicated in drug resistance (Takahashi et al., 2018). TRPC5, involved in ATP-binding cassette subfamily B member 1 (ABCB1)-mediated overexpression of ABCB and cyclin D1, induces nuclear β-catenin accumulation, leading to resistance of gastrointestinal tumor cells to 5-Fluorouracil (5-FU) (Campbell et al., 2018). Increased expression of YY1 has been associated with an anti-apoptotic phenotype in tumor cells (Zhu et al., 2011). Studies suggest that calcium channel blockers, particularly lercanidipine and amlodipine, effectively inhibit YY1. Lercanidipine and amlodipine can be used for targeted and combination therapy in gastric cancer, especially to enhance the efficacy of amphotericin B (Panneerpandian et al., 2021).

**FIGURE 6:**
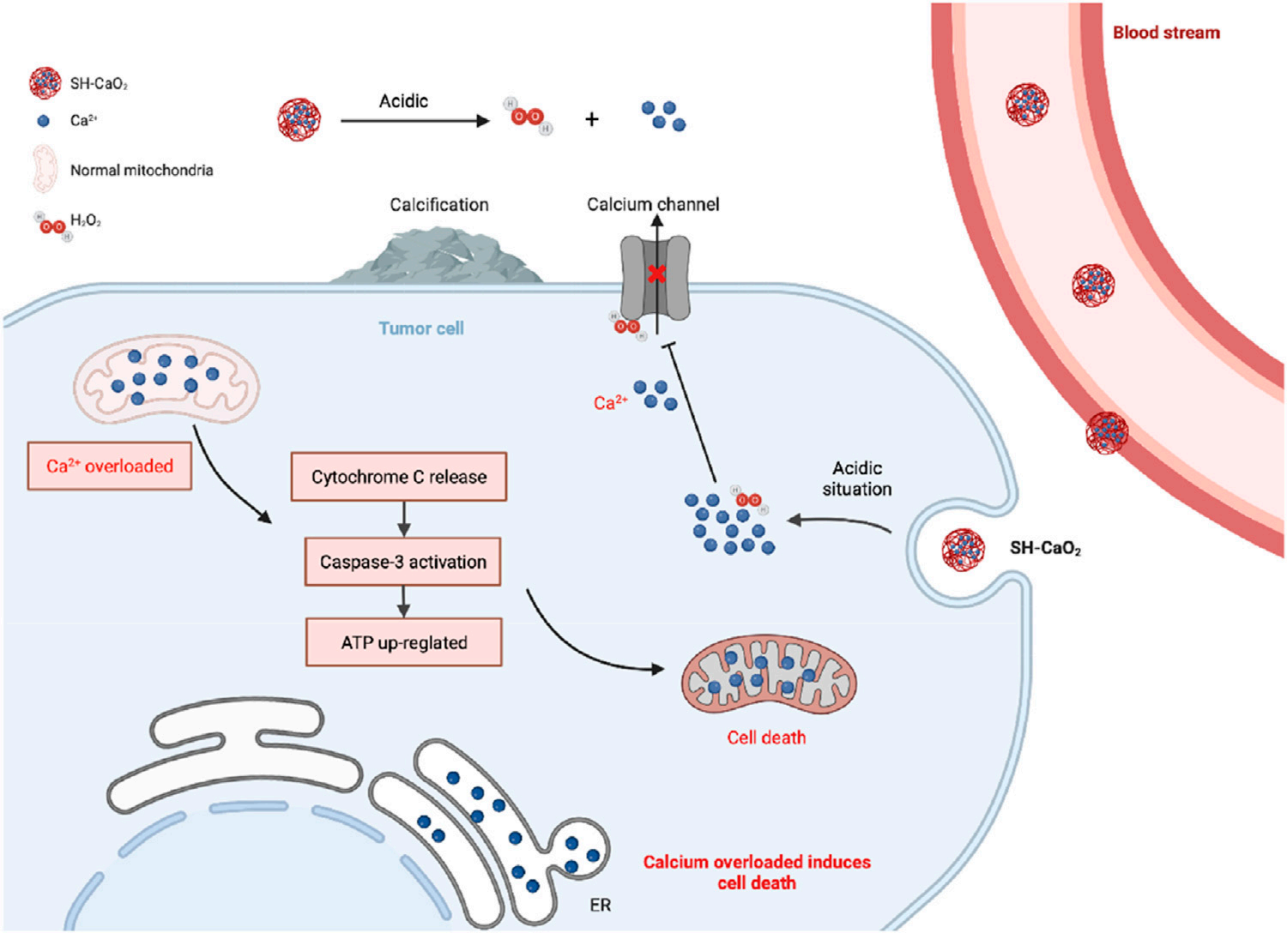
Diagram of calcium overload leading to cell death.

In conclusion, the role of calcium ions in gastric cancer is nuanced yet substantial, manifesting through their ionic state, calcium channels, and calcium-binding proteins. The specific impact on gastric cancer development is contingent on their concentration and expression levels. Ongoing investigations are centered on exploring drug interventions that target these pathways, such as the use of pyrazolo [1,5-a] pyrimidine as a TRPC6 antagonist. *In vitro* experiments have demonstrated its efficacy in inhibiting gastric cancer cell proliferation and migration (Ding et al., 2018), as well as impeding tumor progression in animal models. Moreover, exploring whether TRPC6 antagonists can enhance the effectiveness of common gastric cancer chemotherapeutic agents, including paclitaxel and cisplatin, holds promise (Almasi et al., 2018). Inhibition of TRPM7 has also shown promising results, as it significantly reduces gastric cancer cell proliferation and promotes apoptosis (Kim et al., 2008). Multiple TRPM7 inhibitors have been developed and applied in animal models (Kim et al., 2011; Kim, 2013; Kim et al., 2014), positioning TRPM7 as a promising target for gastric cancer treatment. Furthermore, the potential of inorganic nanomaterials involving calcium ions in tumor therapy is an exciting prospect. Inspired by calcium overload, researchers have successfully synthesized ultra-small SH-CaO_2_ nanoparticles with a diameter below 5 nm, coated with a layer of sodium hyaluronate. In the tumor microenvironment, the protective layer undergoes degradation by hyaluronidase, triggering the rapid decomposition of exposed CaO_2_ and generating abundant H_2_O_2_ and free Ca^2+^ ions. The accumulation of H_2_O_2_ induces oxidative stress, impairing calcium ion channel function and prolonging intracellular retention of Ca^2+^ in tumor cells. Consequently, persistent cellular calcium overload disrupts tumor cell metabolism and functionality, ultimately leading to cell death. Furthermore, localized accumulation of Ca^2+^ promotes calcification in the tumor lesion area (Zhang et al., 2019). These innovative ion interference therapies (IITs) present promising avenues for the clinical application of inorganic nanomaterials in tumor treatment, as depicted in Figure 6.

### 2.7 Magnesium

Magnesium (Mg^2+^), the most abundant intracellular divalent cation in the human body, plays a pivotal role in regulating a multitude of biochemical reactions that impact crucial physiological functions such as nucleic acid metabolism, protein synthesis, and energy production (Jomova et al., 2022). Considering the gastrointestinal effects of magnesium in conjunction with calcium levels is essential. Dai et al. (2013) reported that the recommended calcium-to-magnesium ratio in the diet should be at least 2.63, while Shah et al. (2020) indicated that increasing magnesium intake to >358.9 mg/day in males can reduce the risk of non-cardia gastric adenocarcinoma (NCGA) by 22%–27%. Studies have demonstrated that magnesium ions can modulate several classical pathways, including the Akt/mTOR pathway (Qiao et al., 2019), Wnt/β-catenin pathway (Montes De Oca et al., 2014), and NF-kB pathway (Chen et al., 2022), which are implicated in the development of various cancers, including gastric cancer. Therefore, magnesium ions may play a significant role in regulating the growth, survival, and differentiation of gastric cancer cells, making them a potential target for cancer treatment (Lin et al., 2020). The expression of the classical magnesium ion channel protein, TRPM7, has been observed in human gastric adenocarcinoma (Zou et al., 2019), and inhibiting Mg^2+^ leads to upregulation of TRPM7 expression (Sun et al., 2020) and subsequent inhibition of gastric cancer cell growth and survival (Kim et al., 2014). Similarly, magnesium transport protein SLC41A1 has been shown to play important roles in cancers such as head and neck cancer, breast cancer, and pancreatic cancer (Lin et al., 2015; Uddin et al., 2018; Xie et al., 2019), but its role in gastric cancer remains unexplored. In terms of cancer treatment, supplementing sufficient magnesium may effectively reduce the risk of developing malignant tumors such as bladder cancer, prostate cancer, and colorectal cancer (Yang and Chiu, 1998; Chun-Yuh Yang et al., 2000; Michaud et al., 2000). The mechanism by which it inhibits tumors could be 1) through inhibiting oxidative stress and the consequent oxidative DNA damage that could lead to mutations; 2) by suppressing DNA repair mechanisms to maintain genomic stability (Anastassopoulou and Theophanides, 2002). Clinical reports have demonstrated the analgesic-reducing effects of intravenous magnesium injection (50 mg/kg) during endoscopic submucosal dissection of gastric tumors (Kim et al., 2015). However, the therapeutic effects of magnesium ions or magnesium ion channels themselves on gastric cancer signaling pathways are currently unsupported by evidence. These aspects hold potential as novel therapeutic targets for gastric cancer treatment.

Magnesium is also associated with gastric cancer cell resistance. Human mitochondrial Mrs2 protein (hsaMrs2p), a magnesium transport protein in the inner mitochondrial membrane, is significantly upregulated in multidrug-resistant (MDR) gastric cancer cell lines (Zhao et al., 2002). Studies have shown that the upregulation of hsaMrs2p regulates Mg^2+^ concentration by downregulating p27 and upregulating cyclin D1, while also inhibiting the release of mitochondrial cytochrome C, thereby conferring multiple drug resistance to gastric cancer cells.

In conclusion, magnesium ions, in conjunction with calcium ions, are involved in the occurrence of gastric cancer; however, research on magnesium ions in cancer, especially gastric cancer, is limited, leaving knowledge gaps. Presently, research primarily focuses on magnesium ions as adjuvant therapy in early gastric cancer, but further exploration of magnesium ion channels and transport proteins may unveil promising targets for gastric cancer treatment.

### 2.8 Other trace metals

The human body also relies on trace metal elements such as manganese, cobalt, chromium, and nickel, which exert significant roles in various physiological processes. Studies have shown that abnormal levels of these metals are associated with the occurrence and progression of gastric cancer (Moradi et al., 2015; Annum Afzal and Munir, 2020; Michalke, 2022). Li’s study discovered a direct correlation between elevated manganese levels (>5.12 mg/day) and increased oxidative stress and inflammation, both linked to gastric cancer development (Li and Yang, 2018). Moradi’s research revealed higher manganese ion levels (54%) in gastric cancer patients compared to the control group (42%) (Moradi et al., 2015). In an environmental study conducted in China, prolonged exposure to nickel and its compounds significantly increased the risk of gastric cancer (Chen et al., 2015). A long-term follow-up investigation of diagnosed gastric cancer patients found significantly lower serum nickel levels compared to a healthy control group, potentially related to malignant nutritional depletion (Turkdogan et al., 2022). Chromium, as a heavy metal element, has been a research hotspot for occupational diseases and even cancer caused by occupational exposure. Currently, high-dose chromium exposure or inhalation has been proven to be closely associated with respiratory diseases (including cancer) (Baszuk et al., 2021), although its carcinogenic properties in gastric cancer remain debatable.

These trace metal elements, classified as heavy metals, primarily activate signaling pathways involving reactive oxygen species (ROS) levels. High ROS levels lead to an imbalance in the oxidative-antioxidative system, thereby promoting the formation and progression of gastric cancer (Welling et al., 2015; Li and Yang, 2018). Trace metal elements exhibit similar signaling pathways in the occurrence and development of gastric cancer. Currently, research on the therapeutic effects of trace metal elements in gastric cancer is limited. Cobalt-60, a radioactive element, has been established as a treatment method where it releases specific radiation to eliminate tumor cells. Mature devices such as stereotactic radiosurgery machines are utilized for radiation therapy of various cancers, including gastric cancer (Yahya and Roslan, 2018). However, the therapeutic potential of other trace metal elements in human diseases is still in the exploratory stage.

## 3 The potential of targeting metal in gastric cancer therapy

**FIGURE 7:**
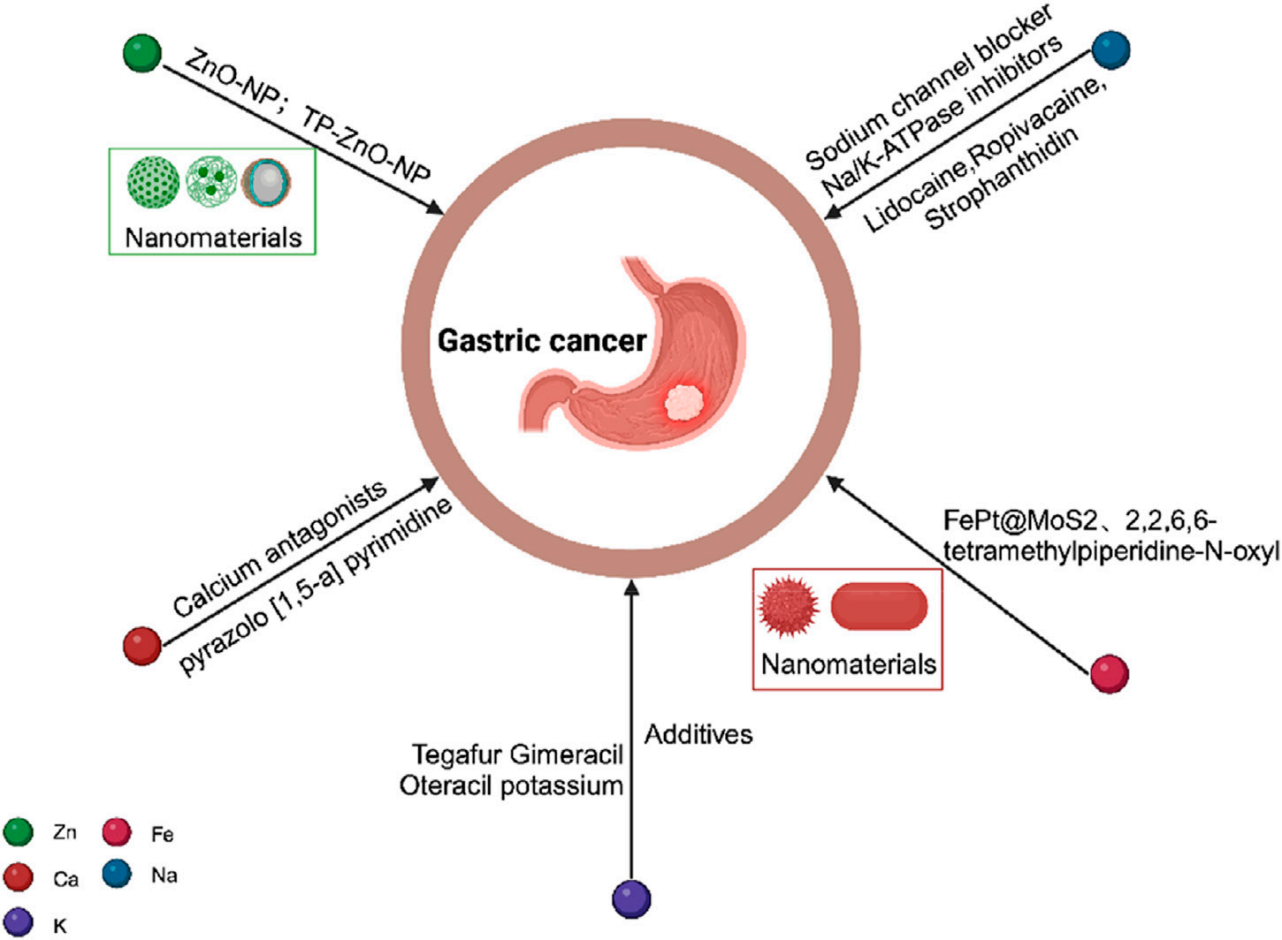
Schematic diagram of potential pathways for metal ion treatment of gastric cancer.

Considering the role of metal ions in gastric cancer, the development of novel nanomaterials and drugs has shown promising therapeutic effects (see Figure 7). For instance, zinc oxide nanoparticles (ZnO-NP) can alleviate gastric cancer cell resistance by inhibiting autophagy (Miao et al., 2021), while another novel nanomaterial TP-ZnO-NP exhibits significant anti-proliferation and anticancer activities against gastric cancer cells (Bozgeyik et al., 2023). Moreover, certain metal ion-based drugs have demonstrated potential in gastric cancer treatment. In the therapeutic domain of gastric cancer, certain sodium channel blockers, such as lidocaine and ropivacaine, have been observed to inhibit the *in vitro* growth of gastric cancer cells (Yang et al., 2018; Ye et al., 2019). Additionally, cardiac glycosides, inhibitors of the sodium-potassium ATPase, have been shown to enhance autophagy in gastric cancer cells by reducing the expression of glucose transporter 1 (GLUT1) (Fujii et al., 2022). Within the realm of cancer treatment ferroptosis, FePt@MoS2 nanoparticles have been established as ferroptosis inducers that release over 72% of Fe(II) within the tumor microenvironment within 30 h of treatment. This expedited release accelerates the Fenton reaction and effectively induces ferroptosis in various cancer cell lines (Zhang et al., 2019). Lactoferrin-iron oxide nanoparticles (LF-IONPs) have also shown remarkable potential in the hyperthermia treatment of gastric cancer (Attri et al., 2023). Additionally, TiO2 nanorods coated with 2,2,6,6-tetramethylpiperidine-N-oxyl, a ferroptosis inhibitor, exhibit the ability to eradicate MCF-7 cell lines while overcoming their multidrug resistance (Fakhar et al., 2020). In the context of gastric cancer treatment, a clinical study involving stage IIb gastric cancer patients and observed that the addition of Huai Er particles (60 mg/day) to Tegafur Gimeracil Oteracil potassium (TGOP, >120 mg/day) significantly improved the prognosis of gastric cancer patients. The study demonstrated that the presence of potassium in TGOP catalyzed the anti-tumor function of Huai Er polysaccharides (Qi et al., 2020), while potassium ions enhanced the cytotoxic effect of chemotherapy drugs on gastric cancer cells by interfering with their uptake (Yeo et al., 2017).

## 4 Discussion

The impact of different metal ions on gastric cancer varies, as they play a crucial role in gastric tissue and mediate various physiological activities. Excessive metal ions under physiological conditions can induce cell death in normal tissues. Different metal ions induce cell death through distinct pathways, such as classical pathways for Na, K, Mg, and Ca ions, and unique pathways for Fe and Cu ions, with other heavy metal ions inducing cell death revolving around ROS. Interestingly, gastric cancer cells exhibit higher resilience compared to normal tissue cells. In terms of the mitochondrial pathway, gastric cancer cells use different mechanisms, such as cellular retinoic acid-binding protein 2 (CRABP2), Ajuba, and MUC20, to maintain mitochondrial homeostasis and resist apoptosis. Gastric cancer cells have developed defense mechanisms against death pathways induced by heavy metals, but their resistance to copper-induced cell death remains an area requiring further investigation. Copper-induced cell death holds promising potential for gastric cancer treatment.

In gastric cancer, there is an imbalance in the concentrations of various metal ions, and metal ion regulation is crucial for gastric cancer cells. For example, excessive intake of sodium ions can promote gastric cancer by creating a favorable environment for the formation of N-nitroso compounds (NOCs). During gastric cancer development, multiple members of Kv channels are upregulated and promote cancer progression through the AKT pathway. Excessive calcium ions enhance the growth and metastasis of gastric cancer through pathways such as calcium-sensing receptor (CaSR) and the AKT/β-catenin pathway. Calcium ion channels are differentially expressed in gastric cancer cells and affect tumor progression through pathways such as Ras/Raf/ERK1/2, JNK, mTOR, and GPCRs. Disruptions in iron ions, whether due to overload or deficiency, promote gastric cancer development. Gastric cancer cells possess mechanisms to resist iron-induced cell death caused by excessive iron ions through signaling pathways involving ALOX15, Wnt/β-catenin, MAT2A-ACSL3, etc. Excessive copper ions in gastric cancer cells increase ROS production and activate pathways such as MAPK, ERK1/2, ULK1/2, promoting tumor progression. Zinc deficiency is associated with poor prognosis in gastric cancer patients. Zinc permeation channels and transport proteins show differential expression in gastric cancer cells compared to normal gastric cells, and they can regulate the biological characteristics of gastric cancer cells through pathways such as MAPK, ERK, JNK.

Drug resistance poses a significant challenge in the treatment of gastric cancer. Different metal ions are involved in inducing resistance in gastric cancer cells. For example, downregulation of potassium channel Kv1.5 expression enhances the resistance phenotype in gastric cancer cells. Calcium ions, through the overexpression of MUC20v2, maintain mitochondrial membrane potential balance, leading to chemoresistance. Overexpression of certain calcium ions such as TRPA1 and TRPC5 promotes chemoresistance in gastric cancer cells. Blocking calcium channels increases the sensitivity of gastric cancer cells to chemotherapy drugs. Upregulation of hsaMrs2p expression in gastric cancer cells leads to increased intracellular Mg^2+^ and upregulation of cyclin D1, resulting in multidrug resistance. Iron ions induce iron-induced cell death and can alter the drug resistance of gastric cancer cells through pathways such as KEAP1/NRF2, STAT3, and Nrf2/Keap1/xCT. Copper ions are mainly involved in gastric cancer cell resistance through changes in copper homeostasis after binding to copper transporter ATP7B and cisplatin, leading to increased resistance to cisplatin. Zinc mainly contributes to gastric cancer cell resistance through zinc finger proteins such as ZFP64, GLI1, ZFP91, ZNF139, which regulate gastric cancer cell resistance through pathways such as GAL-1, MALAT1, AKT-mTOR, or by modulating miRNA. Nanomaterials formed by metal ions, such as Cu, Fe, Ca, Zn, show promise in reversing drug resistance to some extent. For example, Cu can inhibit NF-κB, induce ROS production and autophagy, and induce cancer cell death in novel Schiff base copper coordinated compounds (SBCCCs) (Xia et al., 2019). Ultra-small SH-CaO_2_ nanoparticles can cause calcium overload in tumor cells and induce cell death (Zhang et al., 2019). Applying zinc directly to tumor cells through nanomaterials can also alleviate drug resistance (Miao et al., 2021).

Surgery remains the preferred therapeutic modality for early-stage gastric cancer, embodying the optimal approach. Robot-assisted gastric cancer surgery is poised to establish itself as the leading minimally invasive surgical technique for gastric cancer in the foreseeable future. The management of advanced gastric cancer presents a formidable challenge within the realm of gastric cancer therapy. Notably, the field of immunotherapy for advanced gastric cancer has experienced rapid advancement and has exhibited promising prospects. Anticipated developments in transformative gastric cancer treatments encompass targeted therapies, immunotherapies, NK cell therapies, and combined chemotherapy regimens, all with the ultimate objective of achieving R0 resection. Gastric cancer typically arises due to factors such as *H. pylori* infection, diet, environment, and genetics. Metal ion disturbances, particularly heavy metal ion disorders, are infrequent among the general population, excluding those employed in the metallurgical industry. Nevertheless, an increasing body of research highlights the favorable prospects of employing metal ions in gastric cancer treatment. In conventional chemotherapy, certain ions, such as potassium, have demonstrated their ability to enhance the cytotoxic effects of chemotherapeutic agents on cancer cells. Moreover, heavy metals like cobalt have already found application in routine radiotherapy for gastric cancer. In various *in vitro* experiments, agents inducing iron-mediated cell death as well as iron death inhibitors have proven effective in impeding tumor progression. Copper chelators, including TTM, trientine, and d-penicillamine, have exhibited anti-tumor activity in numerous animal models and clinical trials. Furthermore, the combined utilization of copper ion carriers and copper ions has resulted in elevated intracellular copper levels and exerted anticancer effects. Furthermore, as the mechanisms of various ion channels are investigated in depth and bioinformatics analysis progresses rapidly, certain established drugs can be repurposed for cancer treatment. For instance, tricyclic antidepressants have received FDA endorsement for small cell lung cancer therapy, while aspirin has shown protective properties against colorectal cancer (CRC). Recent reports have emerged regarding the inhibitory effects of verapamil and diltiazem on gastric cancer cells in in vitro experiments, evoking the prospect of novel classical drugs for gastric cancer therapy. Despite the significant challenges in comprehending metal ion-associated signaling pathways and ion channels, particularly in terms of targeting and drug resistance, the targeting of gastric cancer through ion channels and signaling pathways holds substantial promise. Undoubtedly, early diagnosis and effective preventive strategies are paramount in reducing the incidence and mortality rates of gastric cancer. Optimal preventive measures encompass embracing a healthy lifestyle and undergoing regular endoscopic screenings, as they represent the most effective strategies.
